# Psychological Health in Young Adults With Kidney Failure: A 5-Year Follow-up of the SPEAK Study

**DOI:** 10.1016/j.xkme.2023.100637

**Published:** 2023-04-06

**Authors:** Mohammed Al-Talib, Fergus J. Caskey, Carol Inward, Yoav Ben-Shlomo, Alexander J. Hamilton

**Affiliations:** 1 Department of Population Health Sciences, University of Bristol, Bristol, United Kingdom; 2 Richard Bright Renal Unit, Southmead Hospital, North Bristol NHS Foundation Trust, Bristol, United Kingdom; 3 Bristol Royal Hospital for Children, University Hospitals Bristol and Weston NHS Foundation Trust, Bristol, United Kingdom; 4 Exeter Kidney Unit, Royal Devon and Exeter Hospital, Exeter, United Kingdom

To the Editor:

The psychosocial impact of kidney failure in young adults is implicated in the observed high risk of transplant loss^1^ and death^2^ in this group. In the Surveying Patients Experiencing Young Adult Kidney Failure (SPEAK) study, a cross-sectional investigation of psychosocial health among young adults in the United Kingdom aged 16-30 years receiving kidney replacement therapy, we reported worse psychosocial health outcomes among this group than in the age-matched general population.^3^ Using a General Health Questionnaire-12 score cutoff of ≥4 of 12, 31% of young adults had evidence of psychological morbidity compared with 15% among the general population. The specific nature of this psychological morbidity remains unknown, and to our knowledge, there have been no longitudinal studies investigating psychosocial outcomes as this group matures and becomes older. To address this, we undertook the SPEAK-2 study: the 5-year follow-up of the original SPEAK study.

Details of the study population and methods are presented in Item S1. Briefly, individuals recruited to the SPEAK study were invited to complete a revised online survey between June 2020 and January 2021. This included the General Health Questionnaire-12 as well as specific scales to measure symptoms of depression and anxiety: the Patient Health Questionnaire-9 and the Generalized Anxiety Disorder-7 scales, respectively. Patient Health Questionnaire-9 scores of ≥10 of 27 were consistent with symptoms of at least moderate depression. Generalized Anxiety Disorder-7 scores of ≥10 of 21 were consistent with symptoms of at least moderate generalized anxiety disorder. Differences in the baseline characteristics of respondents to SPEAK and SPEAK-2 were analyzed using Pearson’s chi-squared test. Generalized Anxiety Disorder-7 and Patient Health Questionnaire-9 outcomes are reported descriptively. Ethical approval for the study was granted by the Health Research Authority Research Ethics Committee—Brent, reference 20/LO/0534.

We obtained data from 158 individuals (18%; see Fig S1). Those who responded to SPEAK-2 were more likely have a functioning kidney transplant and more likely to be White (Table 1). There was no association between baseline General Health Questionnaire-12 score and response to SPEAK-2 (*P* = 0.1). The proportion of respondents with General Health Questionnaire-12 scores of ≥4/12 increased from 33% to 44% (Table 2). Delineating the nature of psychological morbidity, we found that 40% of respondents had Patient Health Questionnaire-9 scores consistent with at least moderate depression and 35% of respondents had Generalized Anxiety Disorder-7 scores consistent with at least moderate generalized anxiety disorder. Dialysis recipients had worse scores.

**Table 1:** Comparison of the Demographic Characteristics, KRT Modality, and GHQ-12 Scores of Respondents to the SPEAK-2 Versus SPEAK Studies

Characteristics	SPEAK-2 study (n = 158)	SPEAK study (n = 467)	*P* value
	n (proportion)	n (proportion)	
Sex	152	466	0.14
Male sex	70 (46%)	248 (53%)	
Age band	157	496	
<21 y	6 (4%)	105 (21%)	
21 to <26 y	29 (18%)	156 (31%)	
26 to <31 y	54 (34%)	235 (47%)	
≥31 y	68 (43%)		
Median age (y)	30.4	25.7	
Ethnicity	157	444	0.03
White	145 (92%)	368 (83%)	
Asian	6 (4%)	48 (11%)	
Black	4 (3%)	17 (4%)	
Other	2 (1%)	11 (2%)	
Index of multiple deprivation quintile (1 = least deprived; 5 = most deprived)	130	496	0.08
1	21 (16%)	85 (17%)	
2	21 (16%)	104 (21%)	
3	31 (24%)	80 (16%)	
4	21 (16%)	122 (25%)	
5	36 (28%)	105 (21%)	
Current KRT modality	148	465	<0.001
Hemodialysis	16 (11%)	113 (24%)	
Kidney transplant	131 (89%)	323 (70%)	
Peritoneal dialysis	1 (1%)	29 (6%)	
GHQ-12	142	403	
Score of ≥4 of 12 suggesting probable psychological disturbance or mental ill health	63 (44%)	134 (33%)	0.02

*Note:* Data are presented as number and proportion (%). Not all percentages may total to 100 due to rounding. N reported for each characteristic represents the number of respondents for whom data were available. Demographic data for SPEAK study respondents are excluding subsequent SPEAK-2 respondents.

Abbreviations: GHQ-12, General Health Questionnaire-12; KRT, kidney replacement therapy; SPEAK, Surveying Patients Experiencing Young Adult Kidney Failure.

**Table 2:** Psychological Health Survey Results of the SPEAK-2 Study by Current KRT Modality and Overall

Modality	GHQ-12 score ≥4/12 (n = 142)	PHQ-9 score ≥10/27 (n = 122)	GAD-7 score ≥10/21 (n = 121)
	Proportion (95% confidence interval)	Proportion (95% confidence interval)	Proportion (95% confidence interval)
Kidney transplant	41% (33%-50%)	37% (28%-46%)	32% (23%-41%)
Dialysis	71% (44%-98%)	67% (35%-98%)	58% (26%-91%)
Overall	44% (36%-53%)	40% (31%-49%)	35% (26%-43%)

*Note:* Hemodialysis and peritoneal dialysis were combined to account for only 1 SPEAK-2 participant receiving peritoneal dialysis. Scale author recommendations were used for handling of missing data.

Abbreviations: GAD-7, Generalized Anxiety Disorder-7; GHQ-12, General Health Questionnaire-12; KRT, kidney replacement therapy; PHQ-9, Patient Health Questionnaire-9; SPEAK-2, Surveying Patients Experiencing Young Adult Kidney Failure.

Our findings suggest an increased burden of psychological morbidity as young adults with kidney failure age and mature, particularly among dialysis recipients. The reasons for this are unclear. Qualitative studies have explored the psychosocial impact of kidney failure in young adults, with themes of difference and desire for normality, thwarted or moderated dreams and ambitions, and uncertainty and liminality characterizing the lived experience in this group.^4^ Therefore, our findings could represent a long-lasting, pervasive impact of kidney failure in young adulthood upon subsequent life participation. Our findings also characterize the nature of psychological morbidity, with 40% and 35% of respondents having evidence of depression and anxiety, respectively. Although our estimate for the prevalence of depression overlaps with the meta-analytical prevalence of depression among all adults with kidney disease of 34% (95% confidence interval, 31.9%-36.2%),^5^ >90% of individuals in this meta-analysis were receiving dialysis and had higher rates of depression than those seen among transplant recipients. In contrast, 89% of SPEAK-2 respondents had a transplant. Therefore, we may have underestimated the true prevalence of depression. Further longitudinal follow-up will elucidate how the mental health of individuals with kidney failure evolves over their life course.

The survey was conducted during the coronavirus disease 2019 pandemic, and the extent to which this contributed to psychological morbidity is unclear. Psychological health among participants of a longitudinal study of UK households did deteriorate early during the pandemic.^6^ However, this largely returned to prepandemic levels by October 2020, approximately when our survey was active.^7^ This implicates other factors in the observed persistence of poorer outcomes among respondents. One reason could be the impact of shielding, which applied to all dialysis and transplant recipients in the United Kingdom until April 2021 and, in qualitative studies of other high-risk patient groups, had a subjective negative mental health impact.^8^ Longitudinal comparison with the age-matched general population may clarify the true extent and duration of the impact of the pandemic.

There are important limitations to this study. Our response rate was low, particularly among dialysis recipients, which may have introduced bias and limited the generalizability of our findings, as evidenced by broad confidence intervals. The low response rate also meant that we could not analyze the impact of changes in kidney replacement therapy modality on psychosocial health, which may be confounding outcomes.

In conclusion, we characterize a considerable burden of depression and anxiety symptomology among young adults with kidney failure as they age. Further longitudinal follow-up is vital to clarify the impact of the coronavirus disease 2019 pandemic. However, given the known negative impact of poor mental health on adherence, mental well-being,^9^ and employment,^10^ our findings highlight the urgent need for expanded mental health screening, prevention, and treatment for this vulnerable group.

## Supplementary Material

Supplementary File (PDF) Figure S1; Item S1.
