# Supplementary material for: Psychological Health in Young Adults With Kidney Failure: A 5-Year Follow-up of the SPEAK Study
Source: Kidney Med. 2023 Apr 6;5(6):100637. doi: 10.1016/j.xkme.2023.100637 (PMC10248860; doi:10.1016/j.xkme.2023.100637)
Supplement: Supplementary File (PDF) — Figure S1; Item S1. [file mmc1.pdf]

Figure S1 – flow chart of recruitment to SPEAK-2

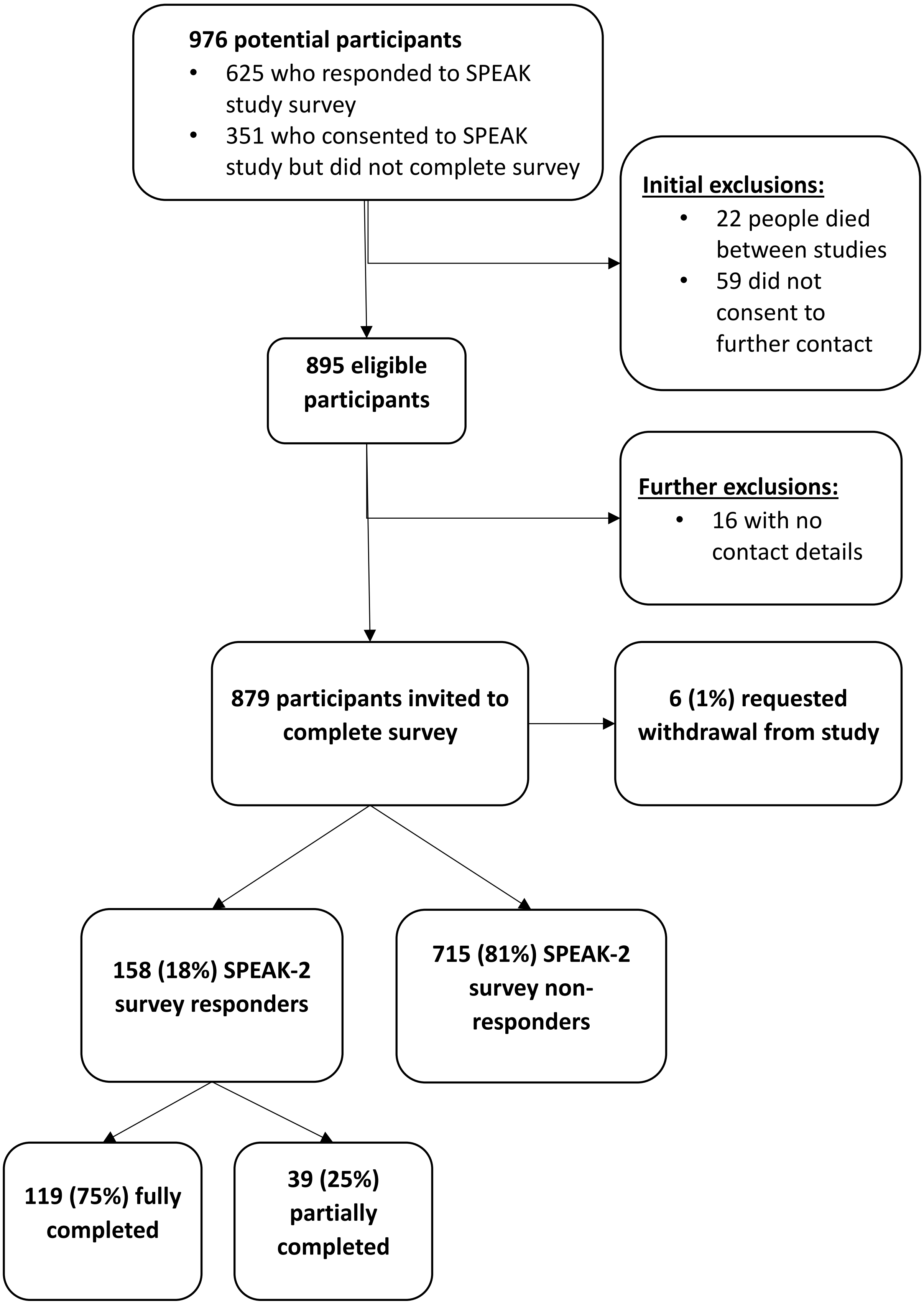

Item S1: Study design and analytical methods

The SPEAK-2 (Surveying Patients Experiencing young Adult Kidney Failure - 2) study is a 5-year follow-up cross-sectional online self-completion survey study of the SPEAK study cohort. The SPEAK study recruited individuals aged 16-30 in the UK receiving RRT between 2015 and 2017. The SPEAK-2 study was granted ethics approval by Health Research Authority National Research Ethics Service Committee Brent, reference 20/LO/0534. The study was funded by a Bristol Health Research Charity (Charity number: 248189) Clinical Research Fellowship (RF08).

Clinical participants and data collection

SPEAK-2 participants were individuals who were recruited to the SPEAK study (n=976) and consented to be contacted for future studies. Of these, 625(64%) participants were respondents to the SPEAK study survey. The remaining 351 (36%) participants had consented to be involved in the study, which allowed for linkage to UK Renal Registry (UKRR) data and obtaining of baseline demographic and clinical information, but had not proceeded to complete the survey. For clarity in our manuscript, a SPEAK 'participant' refers to an individual who consented to the original SPEAK study, whereas a SPEAK 'respondent' refers to an individual who responded to the study survey. We screened SPEAK study consent forms and excluded individuals who did not give consent to be contacted for further studies (n=59). We used linkage with UK Renal Registry (UKRR) data to exclude participants of the original SPEAK study who had since died (n=22), as we did not wish to cause potential distress to their families by sending an invitation to participate in the study. UKRR linkage also allowed us to obtain up to date address information

on potential participants to facilitate invitation to the study. Individuals with missing contact details were also excluded (n=16). A flow-chart of recruitment is presented in Item S2.

A total of 879 eligible individuals were invited to participate via email and/or postal invitation between June 2020 and January 2021. These contained a Patient Information Sheet and a QR code link to the survey. Scanning the QR code directed participants to the e-consent form and, upon completing this, participants were directed to the study survey. Email reminders were sent after 2 weeks and then monthly to individuals who had not started the survey, or who had partially completed the survey. Identifiable information on consent forms (date of birth and postcode) were used to link survey response to those from the original study. Six individuals requested to be withdrawn from the study after receiving an email invitation.

Questionnaire items

Survey items in the original SPEAK study were derived from validated health surveys (HSE and ALSPAC). Using the survey from the original study, we created an online Patient and Public Involvement (PPI) group to help guide elements to keep and sections that were deemed less important. Our discussions were guided by the Standardised Outcomes in Nephrology – Children and Adolescents (SONG-Kids) core outcomes set. For example, as lifestyle behaviours such as smoking and drug use were uncommon in the SPEAK study, these sections were shortened. The PPI group also identified changes to income support in UK since the original survey, so these sections were updated to reflect current provisions.

Two additional scales were incorporated into the survey to characterise the nature of psychological morbidity experienced by this group. The original survey used the General Health Questionnaire-12 (GHQ-12) scale, a screening questionnaire which identifies presence of psychological morbidity but does not define the nature further. Addition of the Patient Health

Questionnaire-9 (PHQ-9) and Generalised Anxiety Disorder-7 scales (GAD-7) allowed us to characterise the nature of psychological morbidity experienced by assessing for symptoms of depression and generalised anxiety disorder respectively. PHQ-9 scores of ≥10/27 are consistent with symptoms of at least moderate depression. GAD-7 scores of ≥10/21 are consistent with symptoms of at least moderate generalised anxiety disorder.

Survey software

Study data were collected and managed using Research Electronic Data Capture (REDCap) hosted at the University of Bristol. REDCap is a secure web-based application designed to support data capture for research studies. It provided greater convenience to our participants than a paper survey and avoided printing, postage, and data entry costs and reduced the risk for introducing data entry errors.

Statistical analysis

We followed scale author recommendations or published methods for handling missing data, using average or lowest-score substitution.

We used Pearson’s chi-squared tests to examine demographic differences between SPEAK-2 (n = 152) respondents and SPEAK respondents who did not respond to SPEAK-2. We excluded SPEAK study responses from those who participated in SPEAK-2 to account for their responses being paired. The analysis of paired psychosocial outcomes are being reported separately. We used univariate logistic regression to examine the association between GHQ-12 score in the SPEAK study and response to SPEAK-2. For the internal comparison of how psychological health changed over time, we used McNemar’s test to compare paired GHQ-12 scores from individuals who responded both to SPEAK and SPEAK-2. PHQ-9 and GAD-7 scores were reported descriptively.

Data cleaning and analysis were undertaken in Stata version 16 (StataCorp LLC).
